# Supplementary material for: Patterns of Intron Gain and Loss in Fungi
Source: PLoS Biol. 2004 Nov 30;2(12):e422. doi: 10.1371/journal.pbio.0020422 (PMC532390; doi:10.1371/journal.pbio.0020422)
Supplement: Table S1 — Also available at http://genes.mit.edu/NielsenEtAl/. (4.3 MB ZIP). [file pbio.0020422.st001.zip › NielsenEtAl/html/1361.html]

AN6601.1.NCU06710.1.MG04052.1.FG05224.1


```
 CLUSTAL W (1.82) Multiple Sequence Alignments - Introns Inserted


Sequence 1: NCU06710.1	657 aa
Sequence 2: MG04052.1	670 aa
Sequence 3: FG05224.1	657 aa
Sequence 4: AN6601.1	633 aa
Alignment Length: 674 aa
Number Identitical Residues: 277 aa
Alignment Score (without introns) 14677


MG04052.1 	MPEGAPAAAGGQAAGQPEQPQE~G0STFRKILQMVAMWVAMQFVMKQFFPANSAPKITTD
NCU06710.1	MPEGNGAQR--QPARQEEQ-QG0S~SMFSKILQAVAMWVLMQLVMKQFMGGGQNTATVQD
FG05224.1 	MAEAQVLPRGQGEQREGGGGGG0G~FSFNKLILGGAIYFGLNAAMNFAMKKDQKGVTVTN
AN6601.1  	MPE---------QRQQREEGEQ~G0SIVRSLIQGVTIFFVTQFFIGQFFGNKQNATAGGK
          	*.*            :       .   . .::   :::.  :  :   :   .      .

MG04052.1 	AD-GKMVEIPTSTGKIPAYHLRPSKLDEGATYNTLPQRVAPIWDEPNKVDVIITVSDSFI
NCU06710.1	AT-GQTVQVSTS---VPSYDQRPQRLDDGTVYNRIPQLIAPIWPENSQVDIIVTISPSFV
FG05224.1 	PDTGEAIVVPGNVEGIPPFELRPKEIFDYLTYREIPKNVAPIWPQDAHVDITVTLSQSFN
AN6601.1  	PG------------AVTSFGERPP-QAEVENYSSVPDTIAPIWPSDSALDISVYVSPSIV
          	.              :..:  **    :   *  :*. :**** .   :*: : :* *: 

MG04052.1 	PTPLSSISKDMIVFEEKDFGIGNFSDKRVAEATIAVPEAVQRNGTLWGHFYVGLPGSVLD
NCU06710.1	PTPLKQTSADKVVFREKKFTIGNWSDARTEERTIDIPTEVQKNGTLWGHFYVGLSGALLD
FG05224.1 	PVPISSVPEEYLVLQEKEFHLSNKTDKRTIETKFTVPPAVQHNGTLWGHFYIGLPGSKLD
AN6601.1  	LPSISSLPSSSLVLEEKKFGLGNYSDVREVETTIQVPKEVQHNGTLWAHFFVALSGHELD
          	  .:.. . . :*:.**.* :.* :* *  * .: :*  **:*****.**::.*.*  **

MG04052.1 	PEQKGFDPATAYHFIYPFTQHIPKKKEKKLKSLLGKKDEAKVDMEDAEEEVEFSGPVVGN
NCU06710.1	PTQPNYDSGSAYHFVWPLTHYIPRKKEFKTRNLLAGKEDAVEEAEEVEVE-QPSGPIITN
FG05224.1 	PKEPGYDTGKAYHFTWPLTQYLPKKKVAKTRNLL---EDMPKHDEEPEEE-EPTGPIIAN
AN6601.1  	PAAKGYSTDTAVHFLRPLNQYLPKRKAKKLKNLLAGADETDEEEDDGTPD-----VSIAS
          	*   .:.. .* **  *:.:::*::*  * :.**.  ::   . ::   :       : .

MG04052.1 	YFHPNMSLSFVPGMGVLGLSQTHVAIRQFLHLEPTGARDGTGQNSWYY~PILFVNTFWQL
NCU06710.1	YYHPNISLSFIPNTGSMPFAQTHPAVRQFLRLEATGARDGTGQNTWYY~PILFVNTFWQL
FG05224.1 	YYHPNASFSFVPDMGVKDFASIAGQMRQYMRLEATGARDRSGQHGWYY1PVLFVNTFWQL
AN6601.1  	YYHPNFTLSVIPDSGNMKYRQIHPAIRRHTQLEATGARDASGQNGWYY~PIVFLNTFWQL
          	*:*** ::*.:*. *     .    :*:. :**.***** :**: *** *::*:******

MG04052.1 	KAHMTQINETVKTLPIRIDLNNLAPWKFQTMASIELSARESARTAAYGGSLPGGGDGTEI
NCU06710.1	KSQMTIINETIKTMPMRIDLNNLAEWKFKTMASIDLSAKEAARAAAFGNPLTTGSDGSEI
FG05224.1 	TNQMTLLNDTVKELPLRIDLNNLASWQFQLMSTMETNSKEAARQAAFGGSLPGGGDGTEI
AN6601.1  	RSHMVELNSTVETIPLRFTLQNLQNWKFAMMASLDENAKQTAKQAAFGGSTPGGGDGSEF
          	  :*. :*.*:: :*:*: *:**  *:*  *:::: .::::*: **:*.. . *.**:*:

MG04052.1 	EMVKEILLDTNPILLAITSVVTVLHLIFETLAFTSDV~QHYRKKKDNIGISVRTILANVF
NCU06710.1	EMIKEILLDSNPILLGVTIFASIAHMILETLAFGSDI~AHYRKKKDNVGISVRSILANVF
FG05224.1 	EMIKEIFLDSNPYLLGVTIVVSILHMILETLAFGSDI~AHYRKKKDNVGISVRSILANVF
AN6601.1  	EMIKEVLLDTNIWLLGTTGVVTILHMIFETLAFKNDI0SHWRKKKDNVGTSVRTILANVF
          	**:**::**:*  **. * ..:: *:*:***** .*:  *:******:* ***:******

MG04052.1 	MQAVIFLYLVDNSQNTSWMILGSQGVGILIELWKVTTVVNVRVRAAPPG---ALIPYRIT
NCU06710.1	MQTIIFLYLVDQSQNTSWMILGSQGVGILIELWKITTVVNVRIRPAP-G---SIIPYRIS
FG05224.1 	MQAVIFLYLLDNSQNTSWMILGSQVVGIVIEFWKITTVVNVRVRPGAPG---SLLPYTIT
AN6601.1  	MQTVIFLYLMDNSENTSWMILASQGFGIVLEAWKVTKTVNVRLRPPPVGSFFSFLPYVVV
          	**::*****:*:*:*******.** .**::* **:*..****:*. . *:  :::** : 

MG04052.1 	FEDKHKLSETEEKTKEYDEIAFKYMYMAGVPLLLAYATYSVIYEEHKSWYSFIITTLVGS
NCU06710.1	FEDKHKLSETEEKTKEYDEIAFKYMYMAGVPLLMAYAIYSLIYDTHKSWYSFVIATLVGS
FG05224.1 	FEDKQKLTETEEKTKEYDEIAFKYMYIAGVPLLIAYGIYSLYYDSHKSWYSYIITTLVGS
AN6601.1  	FEDKHKLTETEKKTQEYDEIAFRYLYIIAVPLLGAYAAYSLMYNTHKSWYSYIIETLVGS
          	****:**:***:**:*******:*:*: .**** **. **: *: ******::* *****

MG04052.1 	VYAYGFLMMVPALYINYRLK~SVAHMPAKAMTYKFLNTFIDDLFAFTIKMPILHRLATFR
NCU06710.1	VYAYGFLMMVPSLYINYRLK~SVAHMPGKAMMYKFLNTFIDDLFAFTIKMPFLHRLATFR
FG05224.1 	VYAYGFLMMIPSLYINYRLK~SVAHMPAKAMMYKFLNTFIDDLFAFTIKMPFLHRLATLR
AN6601.1  	VYAYGFLMMVPSLYINYRLK0SVAHMPGKAMTYKFLNTFIDDLFAFTVKMPWLHRLATLR
          	*********:*:******** ******.*** ***************:*** ******:*

MG04052.1 	DDIIFFVYIYQRWAYRVDYTRVNEFGQGGDDDAPEQVKKDEGKSLPPAVPDVGAPVADSG
NCU06710.1	DDIIFFIYLYQRWAYKVDYTRVNEFGQGGEDDAVEE-KKAEGQKAAFHAPTEEQTVGTT-
FG05224.1 	DDVIFFIYLYQRWAYRIDYTRVNEFGQGGEDEAVDD-KKAKDEAKDKLVEDVKA------
AN6601.1  	DDVIFFIWLYQSYKYKVDYTRVNEFGQGGESDGEEE----EDVKKLDEKENATR------
          	**:***:::** : *::************:.:. ::    :.                  

MG04052.1 	IAKGATGSDARSGKATKRK
NCU06710.1	---SAKASGTETGKAKKRK
FG05224.1 	---EAKTTGSDTGNAKKRK
AN6601.1  	----ATGKQASQTSARKRK
          	    *. . :   .* ***
```
